# Evolutionary and Expression Analyses of the Apple Basic Leucine Zipper Transcription Factor Family

**DOI:** 10.3389/fpls.2016.00376

**Published:** 2016-03-30

**Authors:** Jiao Zhao, Rongrong Guo, Chunlei Guo, Hongmin Hou, Xiping Wang, Hua Gao

**Affiliations:** ^1^State Key Laboratory of Crop Stress Biology in Arid Areas, College of Horticulture, Northwest A&F UniversityYangling, China; ^2^Key Laboratory of Horticultural Plant Biology and Germplasm Innovation in Northwest China, Ministry of Agriculture, Northwest A&F UniversityYangling, China; ^3^College of Horticulture, Qingdao Agricultural UniversityQingdao, China

**Keywords:** apple (*Malus domestica*), bZIP genes, synteny analysis, phylogenetic analysis, gene expression

## Abstract

Transcription factors (TFs) play essential roles in the regulatory networks controlling many developmental processes in plants. Members of the basic leucine (Leu) zipper (bZIP) TF family, which is unique to eukaryotes, are involved in regulating diverse processes, including flower and vascular development, seed maturation, stress signaling, and defense responses to pathogens. The bZIP proteins have a characteristic bZIP domain composed of a DNA-binding basic region and a Leu zipper dimerization region. In this study, we identified 112 apple (*Malus domestica* Borkh) bZIP TF-encoding genes, termed *MdbZIP* genes. Synteny analysis indicated that segmental and tandem duplication events, as well as whole genome duplication, have contributed to the expansion of the apple bZIP family. The family could be divided into 11 groups based on structural features of the encoded proteins, as well as on the phylogenetic relationship of the apple bZIP proteins to those of the model plant *Arabidopsis thaliana* (*AtbZIP* genes). Synteny analysis revealed that several paired *MdbZIP* genes and *AtbZIP* gene homologs were located in syntenic genomic regions. Furthermore, expression analyses of group A *MdbZIP* genes showed distinct expression levels in 10 different organs. Moreover, changes in these expression profiles in response to abiotic stress conditions and various hormone treatments identified *MdbZIP* genes that were responsive to high salinity and drought, as well as to different phytohormones.

## Introduction

Transcription factors (TFs) play key reglatory roles in almost all biological processes, and amongst the several TF families that are present exclusively in eukaryotes, the basic leucine zipper (bZIP) family is one of the largest and most diverse. To date, bZIP family members have been identified or predicted in numerous eukaryotic genomes, including those of plants, animals, and yeast species (Riechmann et al., 2000; Jakoby et al., 2002; Iida et al., 2005; Nijhawan et al., 2008; Yilmaz et al., 2009). They are characterized by a conserved 40–80 amino acid bZIP domain that has two structural features: a basic region that binds DNA and a leucine zipper dimerization motif (Talanian et al., 1990; Hurst, 1994). The basic region is highly conserved and consists of approximately 16 amino acid residues with an invariant N-x7-R/K motif, while the leucine zipper has a less conserved dimerization motif that is composed of a heptad repeat of leucine residues, or other bulky hydrophobic amino acids.

Although members of the bZIP TF family have been identified or predicted in many eukaryotic genomes, relatively few have been functionally characterized. In plants, studies have shown that they regulate physiological processes to provide protection against various biotic/abiotic stresses, such as drought and high salinity, (Xiang et al., 2008; Ying et al., 2012), osmotic stress (Weltmeier et al., 2006), and low temperatures (Shimizu et al., 2005; Liu et al., 2012). They also modulate pathways associated hormone and sugar signaling (Nieva et al., 2005), nitrogen/carbon and energy metabolism (Wang et al., 2011), responses to pathogens (Thurow et al., 2005), and light (Ulm et al., 2004), and are involved in developmental processes that include cell elongation (Yin et al., 1997), organ and tissue differentiation (Silveira et al., 2007), embryonic, and floral development (Zou et al., 2008; Guan et al., 2009), and seed maturation (Jakoby et al., 2002).

The availability of complete genome sequences have allowed genome-wide surveys of bZIP domain genes in plants such as *Arabidopsis thaliana* (Jakoby et al., 2002), rice (*Oryza sativa*; Nijhawan et al., 2008), sorghum (*Sorghum bicolor*; Wang et al., 2011), maize (*Zea mays*; Wei et al., 2012), castor bean (*Ricinus communis*, Jin et al., 2014), cucumber (*Cucumis sativus*; Baloglu et al., 2014), and grape (*Vitis vinifera*, Gao et al., 2014), which have provided insights into their evolutionary and functional relationships. However, such studies have mostly focused on herbaceous plant species, and the bZIP genes of woody economically important fruit tree species have received less attention. Apple (*Malus domestica* Borkh.), also known as orchard apple and table apple, it's one of the most important fruit crops that is widely cultivated in the temperate regions of the world. The genome sequence of the diploid apple variety Golden Delicious was released in 2010 (Velasco et al., 2010), providing an opportunity to identify and analyze protein families at the genome level, and to utilize candidate genes for the genetic improvement of traits such as stress tolerance. In this current study, we performed an analysis of the evolutionary relationships and putative functions of the apple bZIP gene family, including gene identification and analyses of phylogenetic relationships, exon-intron structures, synteny and the expression of selected subset bZIP genes in various tissues, as well as in response to abiotic stresses and hormone treatments.

## Materials and methods

### Identification and annotation of MdbZIP and AtbZIP genes

Representative bZIP TFs were obtained from the iTAK-Plant Transcription factor and Protein Kinase Identifier and Classifier database (http://bioinfo.bti.cornell.edu/cgi-bin/itak/db_browse.cgi). Information about the genes, proteins, and cDNA sequences, as well as the chromosomal location of all the apple bZIP TFs was obtained from the Apple Genome Database (http://www.rosaceae.org/projects/apple_genome). In addition, each protein sequence analyzed using the online program SMART (http://smart.embl-heidelberg.de/; Letunic et al., 2012) to detect the presence of a bZIP domain. The known *A. thaliana* bZIP sequences were downloaded from the TAIR database (http://www.arabidopsis.org/), and the *A. thaliana* protein sequences were annotated according to previously published methods (Corrêa et al., 2008).

### Sequence and phylogenetic analysis

The amino acid sequences of the bZIP proteins of apple and *A. thaliana* were imported into the MEGA5.0 (Tamura et al., 2011) software and multiple sequence alignments were performed using ClustalW. Phylogenetic trees were constructed in MEGA5.0 using the neighbor-joining (NJ) method and a 1000 replicates of the bootstrap test based on the alignment file. The online MEME analysis tool (http://meme.ebi.edu.au/meme/intro.html) was used to identify additional conserved motifs outside the bZIP domain. All 112 MdbZIP protein sequences were used as an input, with the parameters set to optimum motif width ≥6 and ≤200 and the maximum number of motifs to 25 (Jin et al., 2014).

### Exon-intron structure analysis

To obtain information about the intron/exton structures of the apple bZIP TFs, all 112 CDS and the corresponding genomic sequences were analyzed using the Gene-Structure Display Server (gsds.cbi.pku.edu.cn; Guo et al., 2007). This provided a comparison of their CDS with the corresponding genomic sequence, giving both exon position and gene length.

### Synteny analysis and chromosome localization

Synteny blocks within the apple genome and between the apple and *A. thaliana* genomes were downloaded from the Plant Genome Duplication Database (http://chibba.agtec.uga.edu/duplication; Tang et al., 2008) and those containing apple and *A. thaliana* bZIP genes were identified and analyzed. All data used to analyze the expansion patterns of the MdbZIP family are shown in Supplementary Tables S2, S3. Diagrams were generated using the Circos program (version 0.63) (http://circos.ca/). Tandemly duplicated genes were defined as adjacent homologous MdbZIP genes on the same chromosome, with no more than one intervening gene (Zhang et al., 2012). The synonymous (Ks) and non-synonymous (Ka) nucleotide substitutions between orthologous gene pairs were calculated based on the comparative synteny map between the apple and *A. thaliana* genomes, using the ClustalX and PAL2NAL (http://www.bork.embl.de/pal2nal; Suyama et al., 2006) programs to align the protein and coding sequences, respectively.

### Plant material and treatments

*M. domestica* cv. “Fuji” were obtained from an experimental field at the College of Horticulture, Northwest A&F University, Yangling, China (34°20′ N,108°24′ E). Organs, including roots (newly-growing lateral roots of 1–2 mm in diameter), stems (near the apices of newly-growing shoots and of 3–4 mm in diameter), apical buds, flower buds, young leaves (the third to fifth fully expanded young leaves beneath the shoot apices when newly-growing shoots were 40–60 cm in length), mature leaves (leaves on the middle to lower part of newly-growing shoots that were 40–60 cm in length), young fruit (green fruit, approximately 5 cm in diameter), mature fruit (ripe fruit, approximately 10 cm in diameter), and seeds of young fruit and mature fruit were harvested from “Fuji” trees in their adult phase (aged 9–10 years; Li et al., 2015).

For hormone and stress treatments, 2-year-old apple seedlings were used, which had previously been planted in pots, all of which were derived from plants growing in the some conditions. Hormone treatments were performed by spraying young leaves with 300 μM abscisic acid (ABA), 50 μM methyl jasmonate (MeJA), 0.5 g/L ethylene (Eth, released from ethephon), 100 μM salicylic acid (SA), 100 μM gibberellins (GA), followed by sampling at 1, 12, 24, and 48 h post-treatment. As controls for the hormone treatments, leaves were sprayed with sterile distilled water and collected at the same time points (Li J. et al., 2013). Salt stress was applied by irrigating seedlings with 2 dm3 250 mM NaCl (Li X. Q. et al., 2013), and plants irrigated with the same volume of water were utilized as controls. Leaves were collected from both the treated and control plants at 1, 12, 24, and 48 h post-treatment. Drought stress was applied by withholding water from the seedlings, and harvesting leaves at 2, 4, and 7 days post-treatment. Drought-stressed plants were then re-watered to soil saturation, and leaves were collected 2 days after re-watering. For the drought stress experiment, “Fuji” plants watered every 3 days were used as a control, and the leaves were collected at the same time points (Li X. Q. et al., 2013; Li et al., 2015). All plant samples were immediately frozen in liquid nitrogen and stored at −80°C for subsequent RNA isolation and expression analysis.

### Expression analysis of group A MdBZIP genes

Total RNA was isolated from apple samples using the E.Z.N.A.™Plant RNA Kit, according to the manufacturer's instructions (OMEGA, China). Five hundred nanogram DNase-treated total RNA was used for first-strand cDNA synthesis, using a mixture of poly (dT) and random hexamer primers along with PrimeScript RTase (TaKaRa Biotechnology, Dalian, China). The cDNA samples were then diluted and stored at −40°C to be used as a template for the subsequent semi-quantitative RT-PCR and quantitative real-time PCR analyses.

The apple EF-1α gene (GenBank accession DQ341381), amplified with primers F (5′-ATTCAAGTATGCCTGGGTGC-3′) and R (5′-CAGT CAGCCTGTGATGTTCC-3′) was used as an internal control for expression normalization. Other primers (Supplementary Table S5) were designed using Primer Premier 5.0 (Premier Biosoft International), were used to amplify the corresponding CDS. Semi-quantitative RT-PCR was carried out in a reaction volume of 20 μl containing 1 μl of cDNA template, 1.6 μl of gene-specific primers (1.0 μM), 9.8 μl PCR Master Mix (Tiangen Biotech Co. Ltd., Beijing, China), and 7.6 μl sterile distilled water. The PCR parameters were as follows: 94°C for 2 min, 32–40 cycles at 94°C for 30 s, 58–60°C for 30 s, 72°C for 30 s, with a final extension of 72°C for 2 min. PCR products were separated on a 1.5% (w/v) agarose gel with ethidium bromide staining and photographed under UV light. Each reaction was repeated three times and the three independent analyses showed the same trends for each gene and treatment. To validate the semi-quantitative RT-PCR results, real-time PCR analysis was conducted using SYBR green (TaKaRa Biotechnology) on an IQ5 real-time PCR instrument (Bio-Rad, Hercules, CA, USA). Each reaction was carried out in three biological replicates with a reaction volume of 20 μl, including 1.6 μl of gene-specific primers (1.0 μM), 1.0 μl of cDNA, 10 μl of SYBR green (TaKaRa BioInc.), and 7.4 μl sterile distilled water. The PCR parameters were 95°C for 30 s, followed by 45 cycles of 95°C for 10 s, and 60°C for 30 s. The IQ5 software was used to analyze the relative expression levels using the normalized expression method (Hou et al., 2013).

## Results

### Genome-wide survey of the apple bZIP gene family

**Figure 1:**
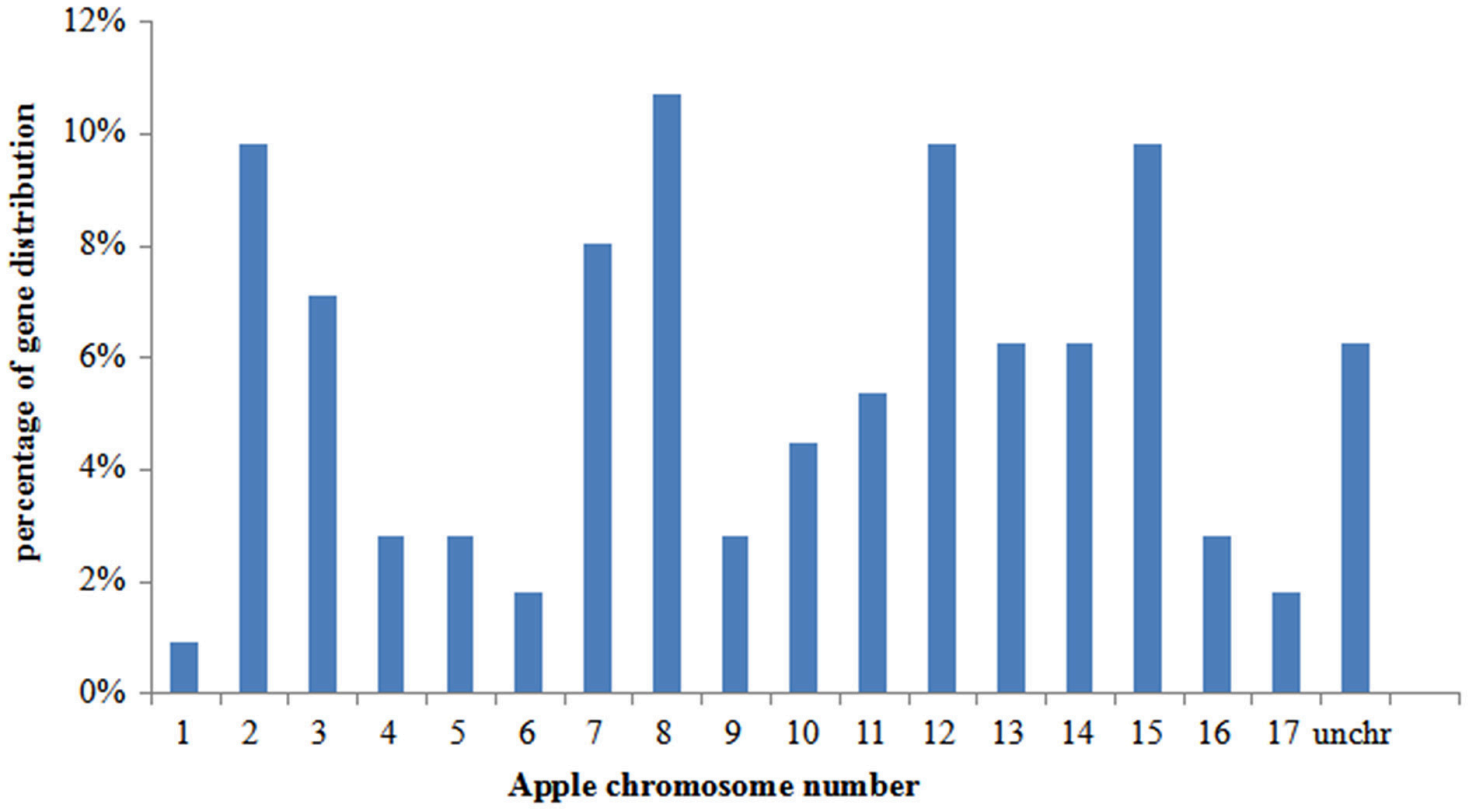
**The distribution of bZIP genes distribution on apple chromosomes, given as percentages**.

A total of 112 putative bZIP genes were identified in a search of the apple Genome Database (Supplementary Table S1), and these were named MdbZIP1 to MdbZIP112 based on their location on chromosomes 1 to 17. Seven bZIP genes (MDP0000120802, MDP0000386314, MDP0000536881, MDP0000545420, MDP0000129203, MDP0000123107, and MDP0000267964) could not be conclusively mapped to any chromosome and were renamed MdbZIP106 to MdbZIP112. The 112 apple MdbZIP genes were found to be distributed between all 17 apple chromosomes, with chromosome 8 containing the highest number (11%), followed by ~10% on chromosomes 2, 12, and 15 and <1% on chromosome 1 (Figure 1). The predicted coding sequence (CDS) and protein size varied substantially between members, with the CDS ranging from 246 to 3459 bp, and the protein length from 100 to 1519 amino acids. The gene names, locus IDs, chromosomal location, CDS and protein lengths are shown in Supplementary Table S1.

### Expansion patterns of the apple MdbZIP family

**Figure 2:**
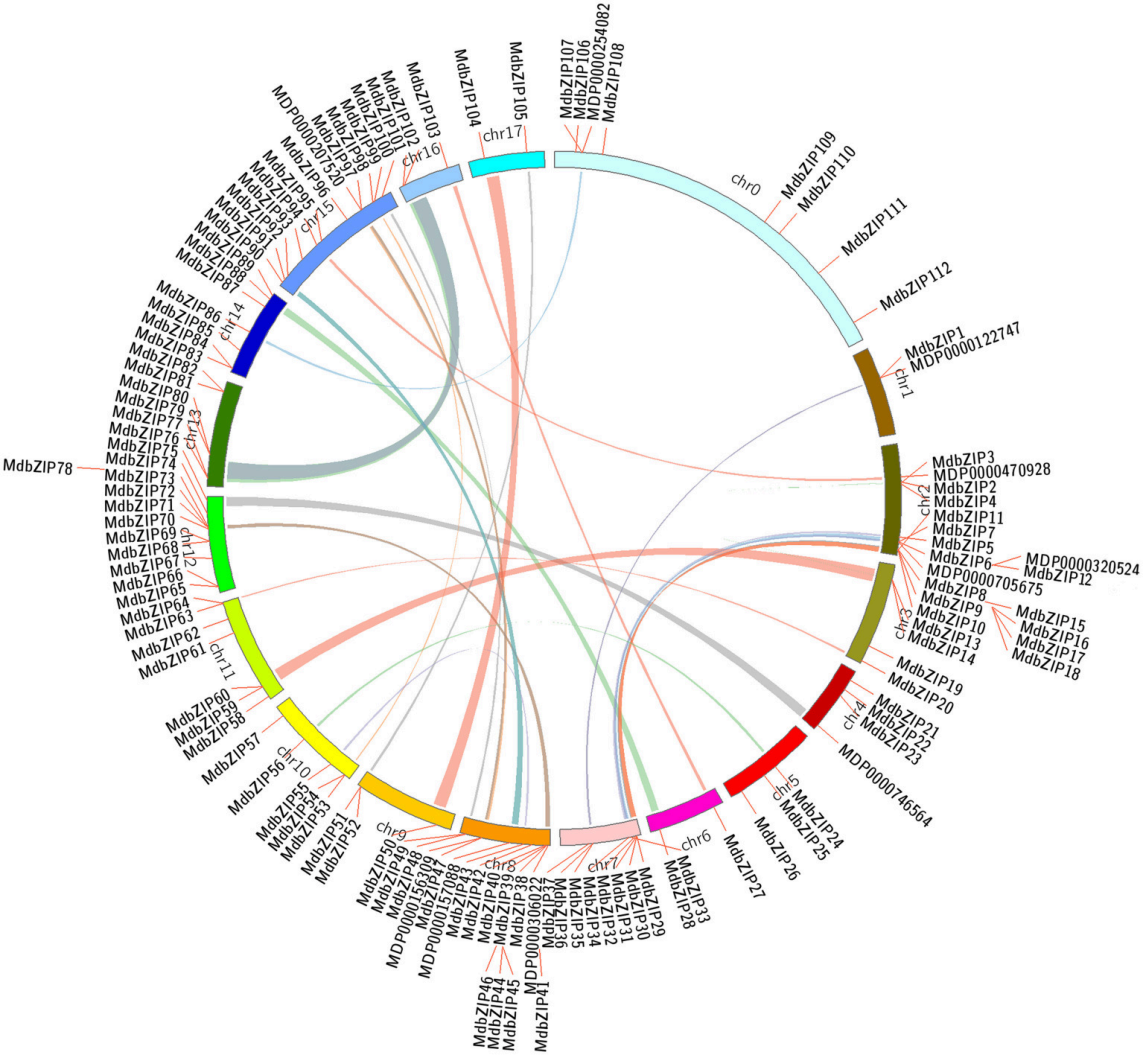
**Distribution and synteny analysis of apple bZIP genes**. MdbZIP genes are indicated by vertical orange lines. Colored bars denote bZIP syntenic regions on the apple genome.

**Figure 3:**
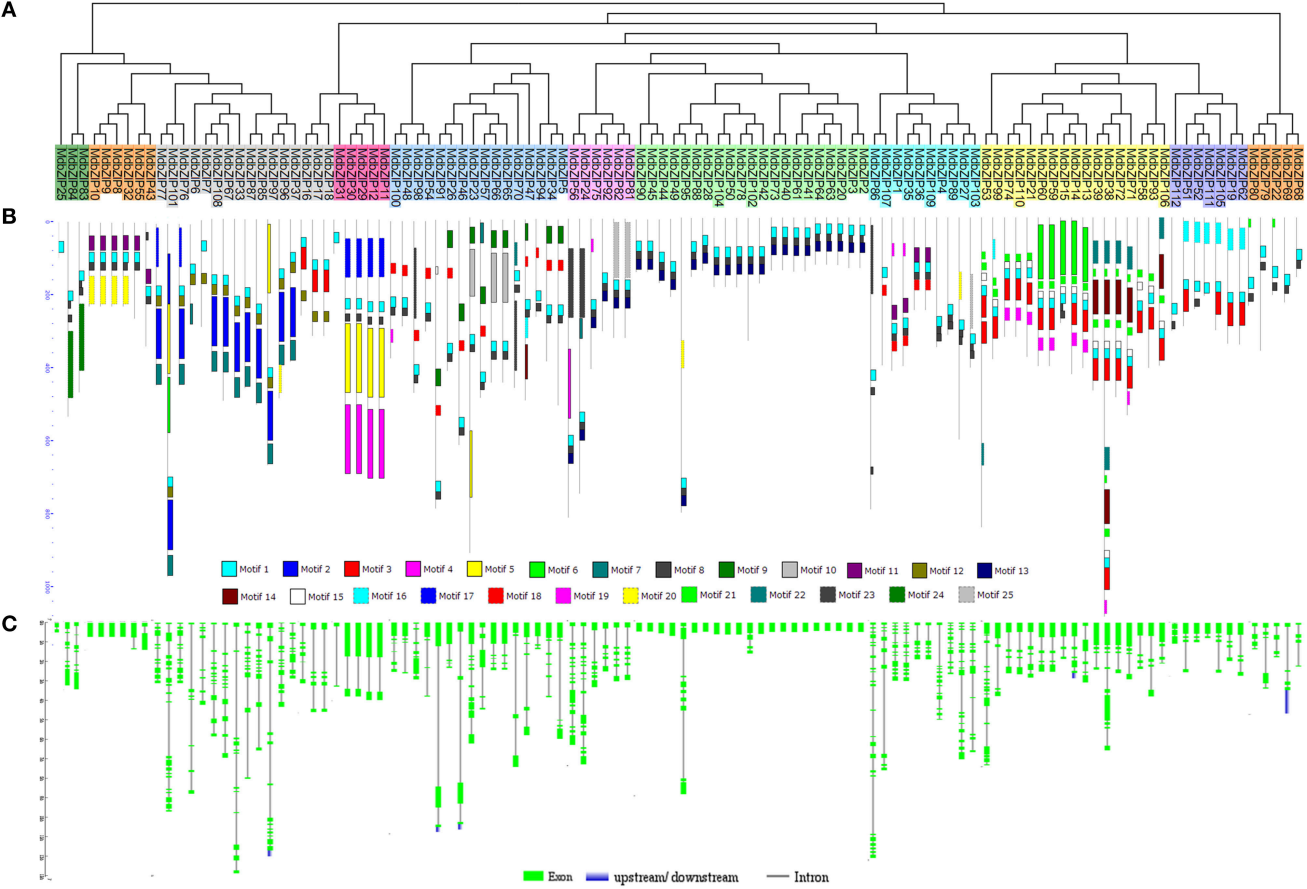
**Characterization of apple bZIP genes. (A)** Phylogenetic analysis of apple MdbZIP proteins. **(B)** Distribution of conserved motifs identified in the 112 MdbZIP proteins. Each motif is represented by a number in a colored box. See Supplementary Table S3 for detailed information regarding the motifs. **(C)** Exon/intron structure of apple bZIP genes, green indicates exons; black indicates introns; blue indicates upstream or downstream.

Segmental and tandem duplications provide two key mechanisms for the expansion of gene families (Cannon et al., 2004) and evidence of both were found in an analysis of the MdbZIP gene family. MdbZIP genes with tandem duplication events were defined as adjacent homologous genes on a single chromosome, while segmental duplications were characterized for its gene duplication events occurred between different chromosomes (Liu et al., 2011), therefore, whether it was regarded as a genes resulting from a tandem duplication event that was depending on their physical location of each MdbZIP gene on its individual chromosome (Guo et al., 2014). As shown in Figure 2, some MdbZIP gene pairs were distributed close to each other on the chromosomes, whose coding sequence lengths and gene structures were similar too (Figure 3); for example, MdbZIP29 and MdbZIP30 on chromosome 7, MdbZIP63, and MdbZIP64 on chromosome 11, and MdbZIP83 and MdbZIP84 on chromosome 14, indicating that they may be tandem duplicated gene pairs. The segmental duplicated blocks within the genome were then compared to the chromosomal location of all the MdbZIP genes. In total, more than 10 pairs of MdbZIP genes, such as MdbZIP76/MdbZIP101, MdbZIP34/MdbZIP5, MdbZIP32/MdbZIP8, as well as others (Supplementary Table S2), were located in pairs of duplicated genomic regions, and most of them were localized to two different chromosomes. There were two exceptions, where the duplicated genomic regions were located to the same chromosome (MdbZIP13/MdbZIP14 on chromosome 3 and MdbZIP2/MdbZIP3 on chromosome 2). In summary, these results suggested that segmental and tandem duplications among the MdbZIP genes may have contributed to the expansion of the gene family.

Interestingly, 9 MdbZIP genes were identified on the chromosomal distribution map (MDP0000122747, MDP0000156309, MDP0000207520, MDP0000470928, MDP0000254082, MDP0000746564, MDP0000705675, MDP0000157088, MDP0000306022) that were not found in our survey, and when these were examined using the online software SMART (http://smart.embl-heidelberg.de/), we found that eight of them (MDP0000122747, MDP0000207520, MDP0000470928, MDP0000254082, MDP0000746564, MDP0000705675, MDP0000157088, MDP0000306022) had no bZIP domains. Rather, these genes were predicted to encode proteins with SCOP, DOG1, DUF4094, or MFMR domains, which were also detected in their corresponding duplicated MdbZIP, and one (MDP0000156309) contained an incomplete bZIP domain.

### Gene structures and phylogenetic relationships of MdbZIP genes

To analyze the evolutionary history the MdbZIP gene family, an unrooted phylogenetic tree was generated using a multiple sequence alignment of predicted MdbZIP proteins (Figure 3A). We observed that bZIP proteins from the same group tended to cluster together that presents a consistency with the two-species analysis (**Figure 5**), while particular genes also displays some differences, such as MdbZIP25, MdbZIP107, and MdbZIP88. To provide further confirmation of the evolutionary relationships among the apple bZIP genes, conserved motifs were predicted using the MEME program (Bailey et al., 2009). In total, 25 motifs were identified (Supplementary Table S3), the distribution of which is shown in Figure 3B. Although the number of amino acids in the apple bZIP TFs varied from 100 to 1519 (Supplementary Table S1), proteins that clustered in the same group tended to share a similar motif composition, which further supported the group definitions. All 112 MdbZIP proteins contained a basic leucine zipper domain (Motif 1), and most had more than one, the exceptions being MdbZIP25 and MdbZIP31. Since exon-intron structure is also known to be an important feature of the evolution of gene families (Zhang et al., 2012; Guo et al., 2014), this was also examined in all 112 MdbZIP genes. As shown in Figure 3C, we identified 23 bZIP genes with no introns, all of which belonged to groups S and F, and which accounted for 21% of the total number of bZIP genes. Among the intron-containing genes, the number of introns in open reading frames varied from 1 to 18 and its intron number had a considerable variation among the different MdbZIP groups. For example, a greater degree of variation in the number of introns was found in groups D and I, varying from 4 to 18 and 1 to 15, respectively, while the number of introns and the variation in number in the remaining groups, such as groups E and H, from 2 to 3 and 3 to 4, was smaller. We therefore, propose that both exon loss and gain occurred during the evolution of the MdbZIP gene family.

### Evolutionary and phylogenetic relationship of apple and *A. thaliana* bZIP TFs

**Figure 4:**
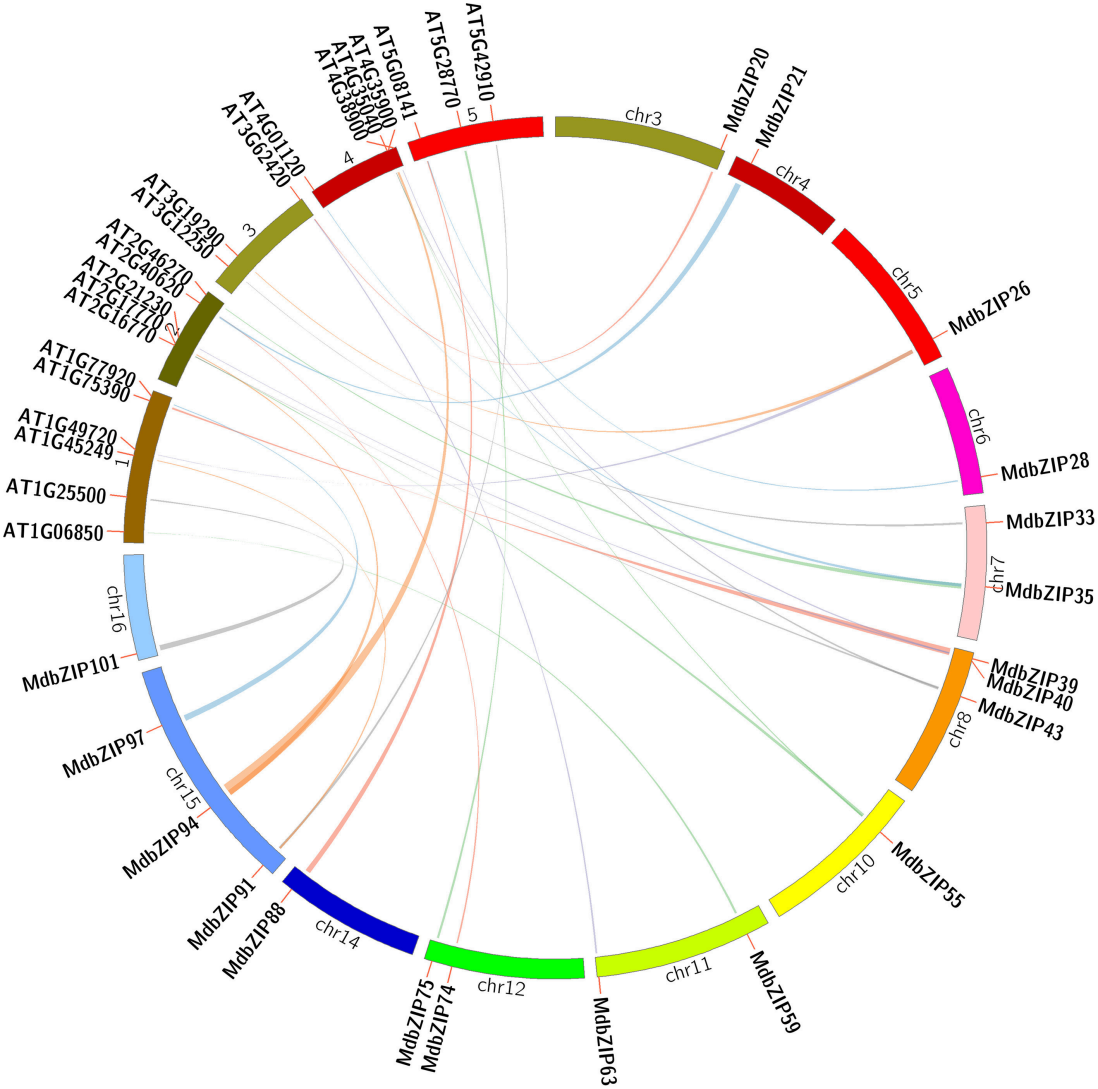
**Synteny analysis of bZIP genes between apple and *Arabidopsis thaliana***. Apple and *A. thaliana* bZIP genes are indicated by vertical orange lines. Colored lines connecting two chromosomal regions indicate syntenic regions between apple and *A. thaliana* chromosomes.

A comparison of the genomes of different organisms can be an effective means to deduce the origin, evolutionary history, and function of un-characterized genes (Lyons et al., 2008). Since *A. thaliana* is the best studied model plant species and the functions of several *A. thaliana* bZIP genes have been well-characterized, we generated a comparative bZIP synteny map of the apple and *A. thaliana* genomes. Large-scale syntenies containing 21 *A. thaliana* and 19 apple bZIP genes were identified (Figure 4 and Supplementary Table S4). Six pairs of syntenic orthologous genes were identified: MdbZIP59-AT1G06850, MdbZIP101-AT1G25500, MdbZIP40-AT1G75390, MdbZIP97-AT1G77920, MdbZIP33-AT3G12250, and MdbZIP75-AT5G28770. In all probability, we propose that these genes are derived from a common ancestor of apple and *A. thaliana*. We noted that, of these, one *A. thaliana* gene (AT1G25500) was not a bZIP gene, but belonged to the plasma-membrane choline transporter family. However, it contained a choline_transpo domain and two trans-membrane regions, which were also detected in MdbZIP101. Interestingly, we also identified five syntenic orthologous gene pairs where a single apple gene corresponded to multiple *A. thaliana* genes: MdbZIP91-AT1G45249/AT5G42910, MdbZIP26- AT1G49720/AT3G19290, MdbZIP94- AT2G17770/AT4G35900, MdbZIP39- AT2G21230/AT4G38900, MdbZIP35- AT2G46270/AT4G01120. Three syntenic orthologous gene pairs with one *A. thaliana* gene corresponding to multiple apple genes were also found: AT2G40620- MdbZIP21/MdbZIP74, AT3G62420- MdbZIP20/MdbZIP63, AT5G08141- MdbZIP28/MdbZIP88. Finally, we detected one case where two apple genes corresponded to multiple *A. thaliana* genes (MdbZIP43/MdbZIP55- AT2G16770*/*AT4G35040). Numbers of synteny events suggested that many bZIP genes arose before the divergence of the *A. thaliana* and apple lineages. For the purpose of evolutionary studies, Ka and Ks values can be used to predict the selective pressure on duplicated genes, such that A Ka/Ks ratio >1 indicates positive selection, Ka/Ks = 1 indicates neutral selection, and Ka/Ks <1 indicates purifying (negative) selection (Krishnamurthy et al., 2015). To explore the divergence of orthologous gene pairs between apple and *A. thaliana*, the Ka, Ks, and Ka/Ks of the orthologous gene pairs were estimated on the basis of the comparative synteny map. Most of the MdbZIP genes had a Ka/Ks ratio <0.2, with the highest found in the AT4G35900-MdbZIP94 pair (Ka/Ks = 0.3082).

**Figure 5:**
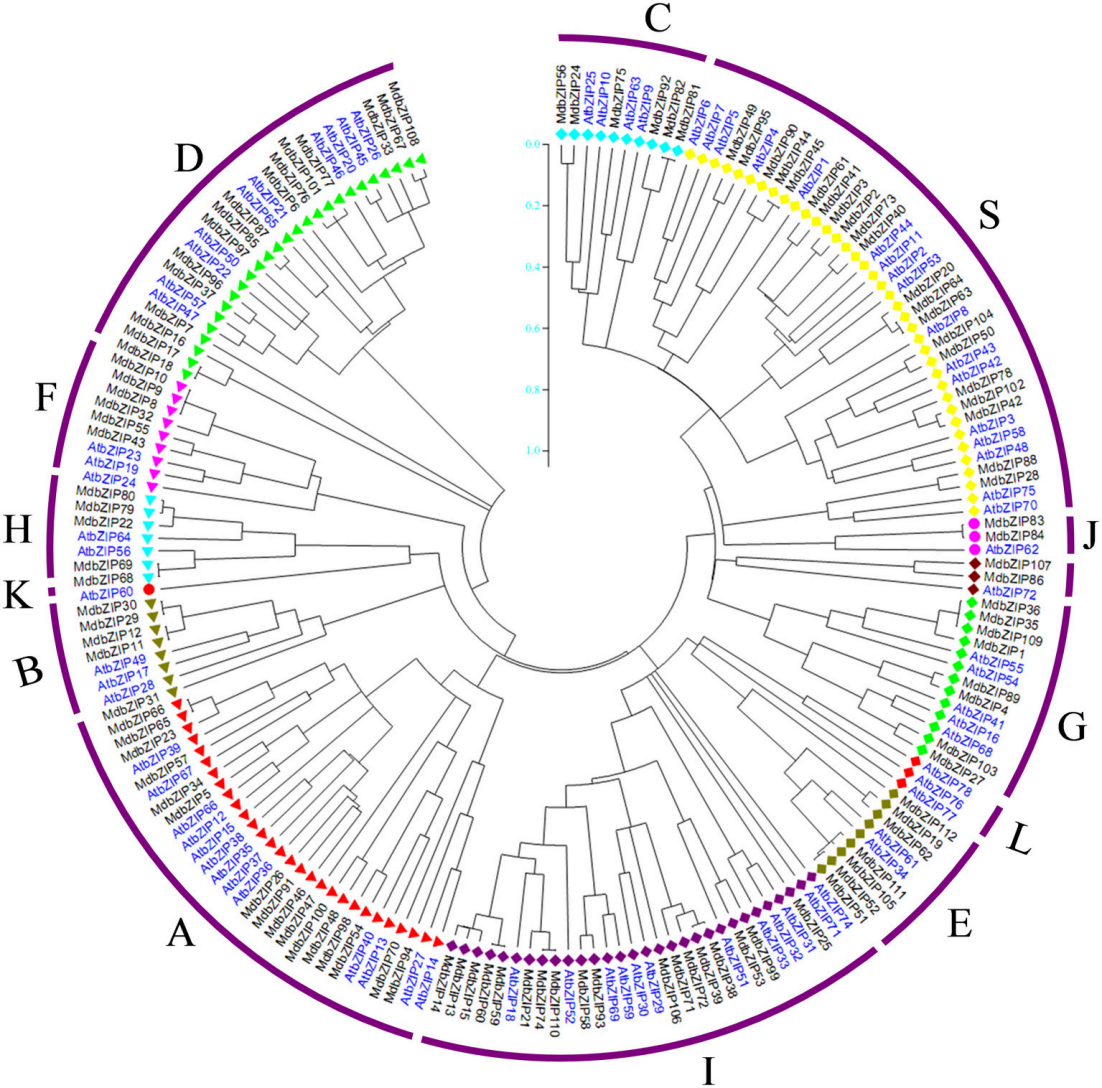
**Phylogenetic analysis (Neighbor joining tree) of apple (black) and *Arabidopsis thaliana* (blue) bZIP proteins**.

An unrooted neighbor-joining (NJ) tree was constructed using the predicted full-length MdbZIP protein sequences and the AtbZIP protein sequences obtained in a previous study (Corrêa et al., 2008; Figure 5). The grouping in apple was found to comply with the classification in *A. thaliana*, and the *A. thaliana* group nomenclature (A, B, C, D, E, F, G, H, I, and S) proposed by Jakoby et al. (2002) was adopted, together with the three extra groups (J, K, and L) classified by Corrêa et al. (2008). Compared to the 13 groups (A, B, C, D, E, F, G, H, I, J, K, L, S) present in *A. thaliana*, which is consistent with Corrêa (Corrêa et al., 2008), 11 groups (A, B, C, D, E, F, G, H, I, J, and S) were found in apple, and bZIP proteins belonging to the same group tended to cluster together in the phylogenetic tree.

### Expression of the apple group A MdbZIP genes in different organs

Group A bZIP genes have generally been associated with abiotic stress regulation, and studies have indicated that group A members function in ABA signal transduction in seeds and vegetative tissues (Jakoby et al., 2002). We analyzed the expression patterns and stress regulation of the 16 Group A bZIP apple genes in order investigate their potential involvement in stress responses. First, we used semi-quantitative RT-PCR to detect their expression in different organs/developmental stages (roots, stems, apical buds, flower buds, young leaves, mature leaves, young fruits, seeds of young fruits, mature fruits, seeds of mature fruits) of plants grown under normal conditions. Most of the genes were expressed in all the tested organs but at varying levels, and they all showed an explicit expression in seeds of both young and mature fruits, except for MdbZIP91, whose expression was weak in seeds of young fruits. The expression levels of one gene varied differently among the 10 organs, example of MdbZIP26, which showed high levels of expression in roots, stems, apical buds, young leaves and mature leaves, but relatively low expression in other organs, and MdbZIP70, which was abundantly expressed in roots, stems and apical buds, but at lower levels in other organs.

### Regulation of the expression of group A bZIP genes by abiotic stress and hormones

We next performed an expression analysis of the group A bZIP genes following exposure to drought, high salinity and hormones, again using semi-quantitative RT-PCR. A range of expression patterns was observed and some of the genes were clearly up-regulated, while others were down-regulated (**Figures 7**, **9**). As shown in **Figure 7**, examples of MdbZIP48, MdbZIP54, and MdbZIP98 showed a significant increase in transcript levels after the plants were sprayed with the hormone abscisic acid (ABA), while the expression MdbZIP5 and MdbZIP91 decreased and MdbZIP66 transcript levels decreased 1 h post ABA treatment, but then increased. Ethylene treatment caused MdbZIP48 and MdbZIP66 transcript levels to increase, while MdbZIP47 and MdbZIP98 exhibited decreased expression at least at 12 and 48 h after treatment. MdbZIP34, MdbZIP48, and MdbZIP66 showed an obvious increase in expression from 12 to 48 h post-treatment with methyl jasmonate (MeJA), and MdbZIP26, MdbZIP66, MdbZIP70, MdbZIP100 transcript levels increased at least at 12 and 24 h after treatment with salicylic acid (SA). Two genes, MdbZIP34 and MdbZIP48, showed an increase in expression at different time points following the SA treatment, but none of the genes showed a decrease in expression. Only MdbZIP47 showed a clear decrease in transcript levels at all time points after treatment with gibberellins (GA), while MdbZIP66 and MdbZIP91 both showed increased expression.

Examples for transcript levels of MdbZIP5 (48, 96, 168, and R48 h) and MdbZIP70 (48 and R48 h) increased in response to drought (**Figure 9**), while MdbZIP54 expression decreased at all time points. MdbZIP47 and MdbZIP98 both showed lower expression following the application of salt stress. In addition, when exposed to drought, the expression of some genes, such as MdbZIP66, MdbZIP70, MdbZIP100, decreased gradually with time while restored after the plants were re-watered. Finally, some group A bZIP genes showed no appreciable change in expression following either abiotic stress or hormone treatment, compared to the control, such as genes of MdbZIP5 under methyl jasmonate (MeJA) treatment, MdbZIP70 under gibberellins (GA) treatment, MdbZIP34 following drought stress, and so on.

To verify the efficacy of the semi-quantitative RT-PCR analyses, the expression of three randomly selected bZIP genes (MdbZIP26, MdbZIP47, and MdbZIP48) following abiotic stress and various hormone treatments was also determined by quantitative real-time PCR (Figures 8, **10**). These results of the two approaches were generally consistent.

## Discussion

### Expansion and synteny of the apple bZIP family

**Figure 6:**
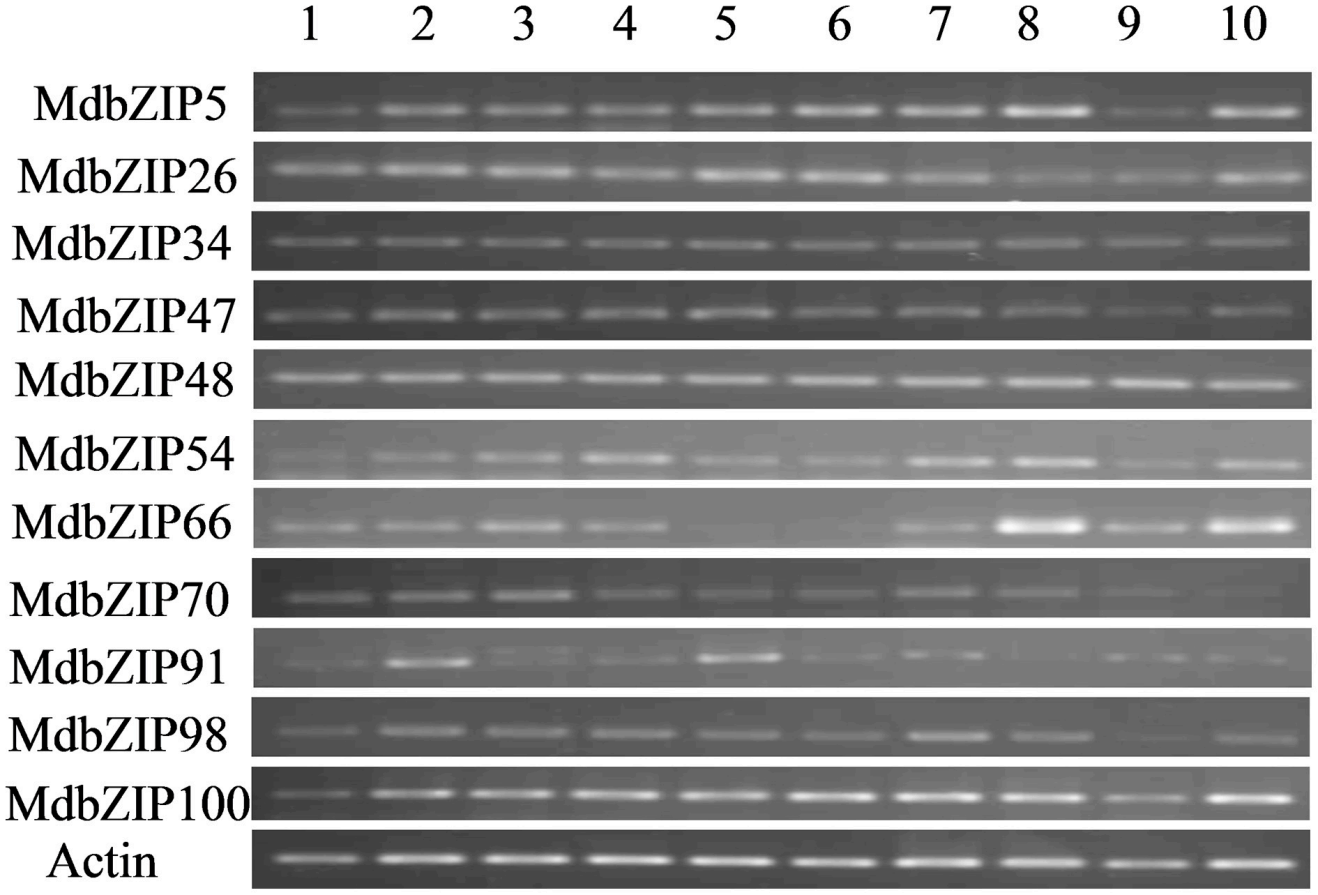
**Expression analysis of the 11 MdbZIP genes from group A in various organs, as determined by semi-quantitative RT-PCR analyses**. Organs are indicated as follows: 1-roots, 2-stems, 3-apical buds, 4-flower buds, 5-young leaves, 6-mature leaves, 7-young fruits, 8-seeds of young fruits, 9-mature fruits, 10-seeds of mature fruits.

**Figure 7:**
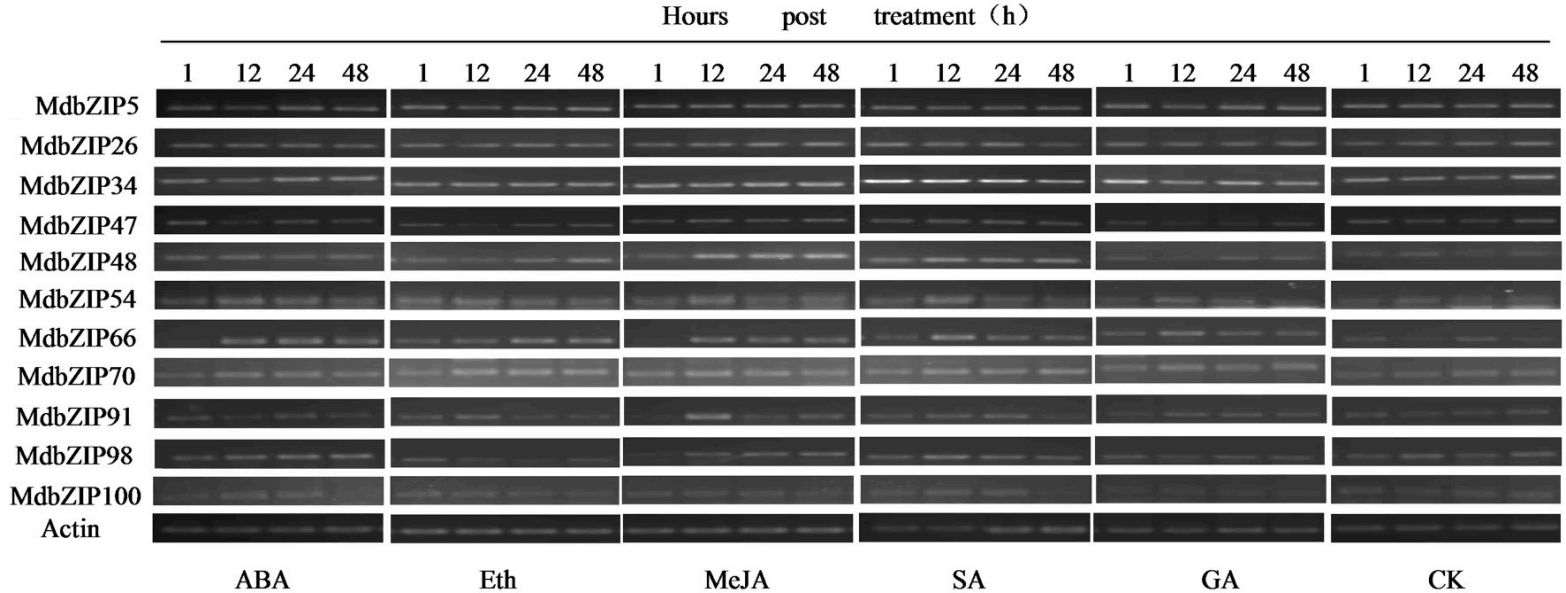
**Expression patterns of apple group A bZIP genes following hormone (ABA, Eth, MeJA, SA, GA) treatments**. Expression patterns of MdbZIP genes following treatment with 300 μM ABA, 0.5 g/L Eth, 50 μM MeJA, 100 μM SA, and 100 μM GA, as determined by semi-quantitative RT-PCR analysis. For each gene, five bands on the left side represent amplified products from the treated leaves, one band on the right represent amplified products from untreated leaves. The apple *EF-1a* gene was used as the reference gene. Hours (1, 12, 24, 48) post-treatment are indicated. ABA, abscisic acid; Eth, ethylene; MeJA, methyl jasmonic acid; SA, salisylic acid; GA, gibberellins.

Gene duplication, including tandem, segmental, and whole genome duplication, has played an important role in the evolution of organisms (Xu et al., 2012), and land plants have undergone abundant gene duplication throughout their evolutionary history (Doyle et al., 2008). Based on a comprehensive analysis of the MdbZIP genes, we concluded that segmental and tandem duplications have played important roles in the expansion of the MdbZIP gene family, which is consistent with previous studies of other species. It was concluded for cucumber (Baloglu et al., 2014), rice (Nijhawan et al., 2008), grape (Gao et al., 2014), and maize (Wei et al., 2012) that segmental chromosomal duplication is one of the expansion mechanisms and that has contributed most to the bZIP family enlargement, and more so than tandem duplication. Whole-genome duplication events (γ, β, α) are a common phenomenon in angiosperms (Zhang and Wang, 2005) and often lead to gene family expansion (Cannon et al., 2004). It has been reported that genome duplication expands genome content and diversifies gene functions, driving plant adaptability, and evolution (Li J. et al., 2013). It was also reported that a relatively recent (>50 million years ago) whole genome duplication has contributed to the formation of the 17 chromosomes in apple, which were derived from nine ancestral chromosomes (Velasco et al., 2010). Since members of this bZIP family regulate so many physiological and biological processes, including responses to biotic/abiotic stresses and signaling during growth and development, it is likely that geneome duplication also may have contributed to the expansion of the MdbZIP gene family in order to diversify gene function and plant adaptability. According to Zhang (2003), not all duplication events are stable. During selection and evolution, duplicated genes can be can be stably maintained when they differ in some aspects of their functions (Nowak et al., 1997), which may be the reason why the segmental duplicated gene pair MdbZIP98/MdbZIP54 from group A have different expression levels in response to both abiotic stresses and hormone treatments as well as different expression pattern in various organs (Figures 6, 7, 9).

**Figure 8:**
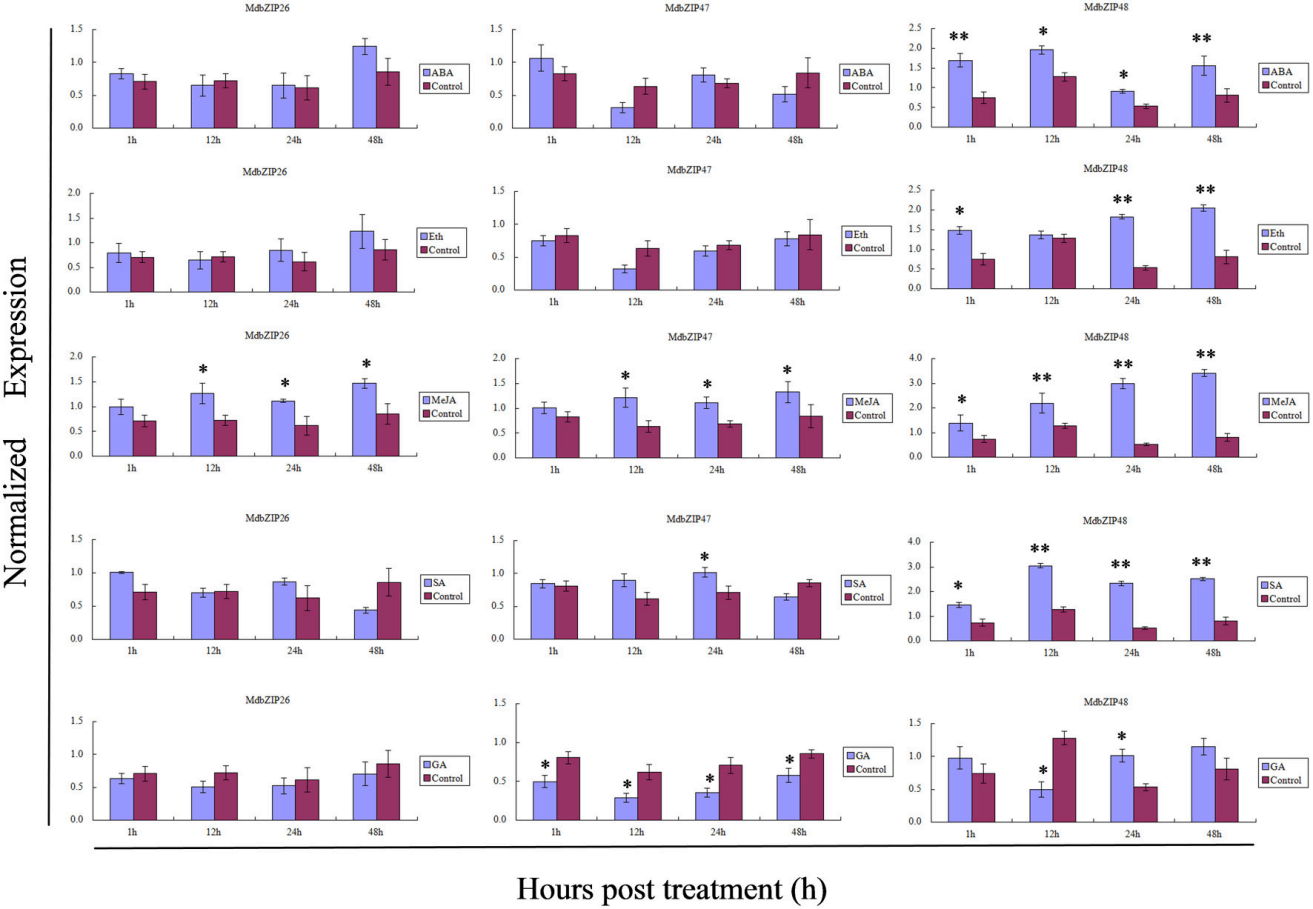
**Transcript levels in leaves of three randomly selected MdbZIP genes (MdbZIP26, MdbZIP47, MdbZIP48) following treatments with abscisic acid; ethylene; methyl jasmonic acid; salisylic acid; gibberellins., determined by quantitative real-time RT-PCR**. The apple *EF-1a* gene was used as an internal control to normalize the data. The error bars were calculated based on three biological replicates. The data are shown as the mean values ± SD. ^*^ and ^**^indicate the corresponding gene signifcantly up- or down-regulated under the differential treatment by *t-*test (^*^P < 0.05, ^**^P < 0.01).

**Figure 9:**
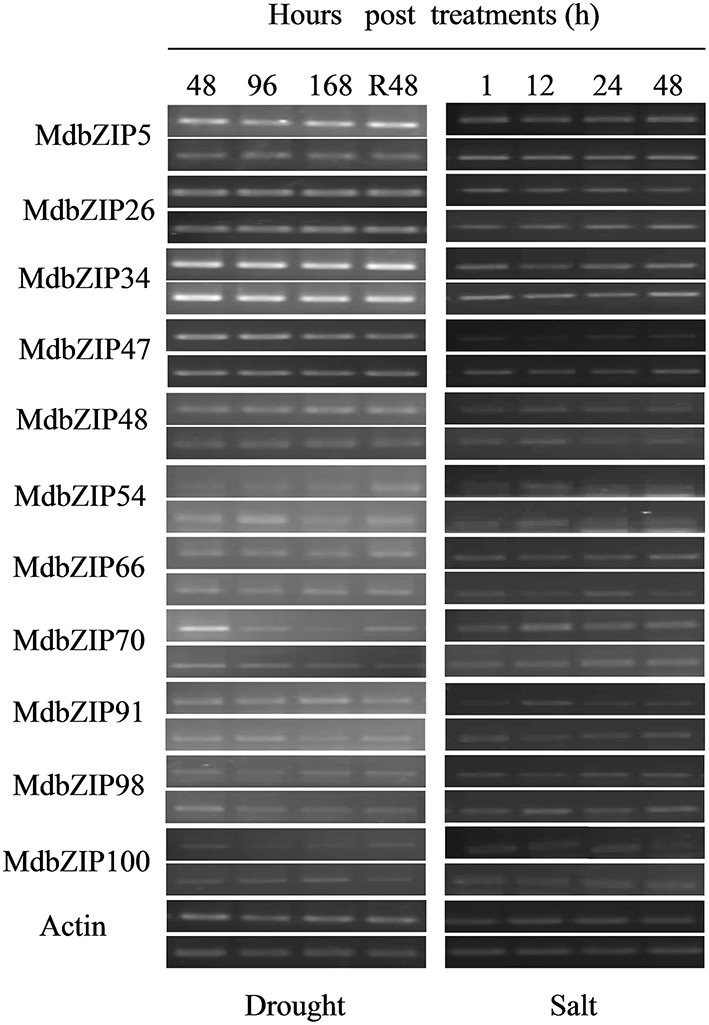
**Expression patterns of apple bZIP genes from group A following abiotic stress treatments (drought and saline conditions)**. Expression patterns of MdbZIP genes following drought and salt treatment were determined by semi-quantitative RT-PCR analyses. For each gene, the upper band represent amplified products from the treated leaves, band under them represent amplified products from untreated leaves. The apple *EF-1a* gene was used as the reference gene. Hours (1, 12, 24, 48) post-treatments are indicated in the graph.

### Structural and phylogenetic analysis of apple bZIP proteins

Since exon/intron structure can also provide important evidence to support phylogenic relationships in a gene family (Li et al., 2006), we investigated the exon/intron organization of the 112 MdbZIP genes to obtain further insight into their possible structural evolution, since exon/intron diversification of gene family members is known to have played an important role in the evolution of multiple gene families (Xu et al., 2012). We determined that ~20% of the genes had no introns, which is similar to the percentages observed in rice and sorghum (both 19%; Nijhawan et al., 2008; Wang et al., 2011). We found that the two genes in a duplicated gene pair tended to be clustered into one group, when phylogenetic analysis was performed, such that tandem pairs MdbZIP83-MdbZIP84 (7 exons) and MdbZIP63-64 (without introns) were clustered to group J and S, respectively. This was also true for the segmentally duplicated pairs. Some duplicated genes had the same number of exons and nearly identical lengths, indicating that segmental or tandem duplication expanded the bZIP family.

Exon/intron gain/loss is one of mechanism for diversification of multiple gene families (Wang et al., 2015), and was also observed in this study. For example, MdbZIP27 contained 16 exons, while the paralogous gene, MdbZIP103, had only 14 (Figure 3), indicating a loss of 2 exons during evolution, which is a similar pattern to that reported for the grape family (Gao et al., 2014). These gain/losses may be the results of chromosomal rearrangements and fusions, and can potentially lead to the generation of functionally distinct paralogs (Xu et al., 2012; Guo et al., 2013). In contrast, genes clustering together generally possessed similar exon/intron organization, showing a correlation between the phylogeny and exon/intron structure. This also correlated with the predicted motif analysis, where proteins clustered in the same group tended to share similar motif composition, a pattern that has also been observed in grape (Gao et al., 2014).

### The evolution of bZIP proteins in apple and *A. thaliana*

Comparative genomics approaches structure genomes into syntenic blocks that exhibit conserved features across the genomes (Ghiurcuta and Moret, 2014), the synteny analysis provides evolutionary and functional connections between apple and *Arabidopsis* syntenic genes (Zhang et al., 2015). The roles of many *Arabidopsis* genes in plant abiotic stress responses have been extensively studied recently. For example, AtbZIP36 (ABF2/AREB1), AtbZIP38 (ABF4/AREB2), and AtbZIP37 (ABF3) genes were up-regulated in response to the aBa signal, dehydration, and salinity (Uno et al., 2000). AtbZIP11 (ATB2) might play an important role in the sugar signaling pathway during metabolism (Rook et al., 1998). In this study, both syntenic and phylogenetic analyses was performed to assess the relationship to the model plant, *A. thaliana*, and to infer the functions of orthologous genes. A total of 21 *A. thaliana* bZIP genes and 19 apple genes were identified as syntenic orthologs. Of these, six pairs appeared to be single apple-to-*A. thaliana* pairs, indicating that these genes were likely present in the genome of the last common ancestor of these two species. The remaining gene combinations showed a more complex relationship, with five gene-pairs that single apple genes corresponding to multiple *A. thaliana* genes, three single *A. thaliana* genes corresponding to multiple apple genes, and one case where two apple genes corresponded to multiple *A. thaliana* genes. Since some of the bZIP genes present in these two species that could not be mapped into any syntenic blocks, it could not be determined whether these share a common ancestor, this may be explained by the fact that after the speciation of apple and *Arabidopsis*, their genomes have undergone multiple rounds of significant chromosomal rearrangement and fusions, followed by selective gene loss, which can severely obscure the identification of chromosomal syntenies (Paterson et al., 2012; Zhang et al., 2012; Gao et al., 2014). In summary, we conclude that some of the apple genes appeared to share a common ancestor with their *A. thaliana* bZIP counterparts, and since all the Ka, Ks, and Ka/Ks of the syntenic orthologous gene-pairs had Ka/Ks ratios <1, they likely underwent a strong Darwinian purifying positive selection.

Based on phylogenetic analysis, the apple MdbZIP proteins could be divided into 11 groups, and the *A. thaliana* proteins into 13 groups. No species-specific groups were observed, suggesting evolutionary conservation amongst the bZIP TFs. bZIP proteins from the two species belonging to the same group tended to cluster together in the phylogenetic tree, suggesting that they may have experienced duplication after the lineages diverged. In addition, most syntenic orthologs from apple and *A. thaliana* tended to be clustered into one group in the phylogenetic tree, suggesting that prediction of MdbZIP function can be made on the basis of the reported functions of the *A. thaliana* homologs, while further experimental evidence are required to deeply understand their biological roles and the pathways they involved in.

### MdbZIP genes play important roles in physiological and biological processes

**Figure 10:**
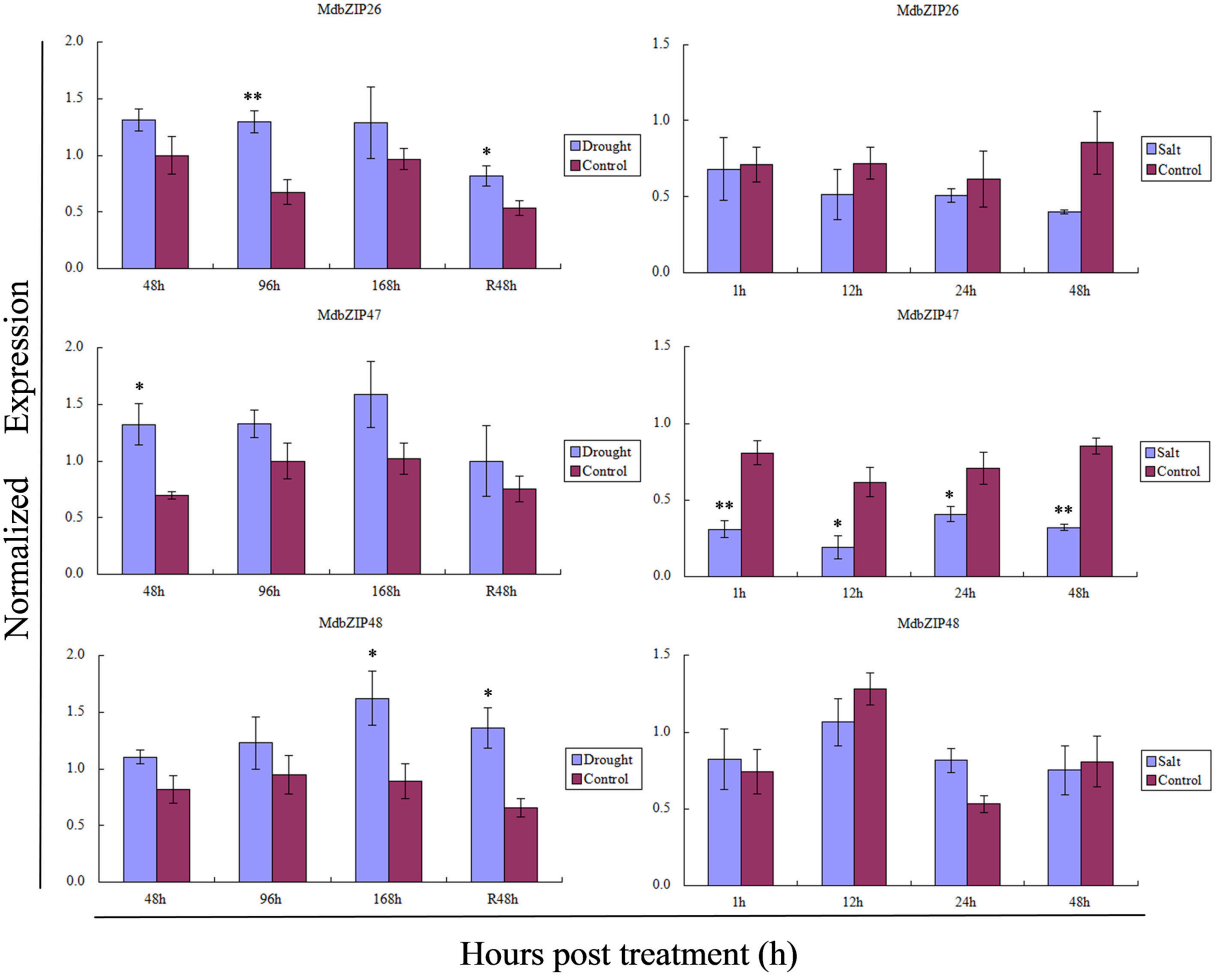
**Transcript levels in leaves of three randomly selected MdbZIP genes (MdbZIP26, MdbZIP47, MdbZIP48) following drought and salinity stress treatments, determined by quantitative real-time RT-PCR**. The apple *EF-1a* gene was used as an internal control to normalize the data. The error bars were calculated based on three biological replicates. The data are shown as the mean values ± SD. Asterisks represents the corresponding gene signifcantly up- or down-regulated under the differential treatment by *t*-test (^*^P < 0.05, ^**^P < 0.01).

There is considerable evidence that bZIP genes are master regulators of many central developmental and physiological processes (Alves et al., 2013), including involvement in multiple biological processes and response to abiotic and biotic stresses (Sornaraj et al., 2016). The function of group A bZIP genes also had been studied in other plant species, especially in *A. thaliana*. For example, AREB1 was reported to enhance drought stress tolerance in *A. thaliana* (Fujita et al., 2005) and overexpression of SlAREB1 play a role in abiotic stress response in cultivated (*Solanum lycopersicum*) and wild (*Solanum* spp.) tomato species (Yáñez et al., 2009). Kang et al. (2002) reported that overexpressing of another group A member ABF3 in *Arabidopsis* exhibited reduced transpiration and enhanced drought tolerance. In addition to regulating stresses, group A bZIP genes also function in seed development, responses to ABA and fruit maturation (Corrêa et al., 2008). Based on the expression data (Figure 6) all investigated apple organs expressed at least one group A bZIP gene, indicating that they may play an extensive role in apple development. Most of the expressed genes were detected to some degree in roots, stems, apical buds and leaves, as well as in young and mature seeds, which is consistent with previous studies in *A. Thaliana* (Jakoby et al., 2002). It is worth noting that the expression of MdbZIP66 in leaves was very weak under normal growth conditions, but higher following stress and hormone treatments (Figures 7, 10), while the expression of five genes (MdbZIP23, MdbZIP46, MdbZIP57, MdbZIP65, and MdbZIP94) was not detected by RT-PCR in any organ, even in response to drought or exogenous hormone applications. A similar circumstance was observed in grape (Gao et al., 2014) and sorghum (Wang et al., 2011), and we hypothesize that these genes may have been silenced or that their expression is too weak to detect. Another possibility is that they respond to other abiotic/biotic factors or that they are not annotated correctly (Wang et al., 2011; Gao et al., 2014), or they may be expressed at other organs and time point after hormone and abiotic treatment.

It has been reported that plant hormones play important roles in diverse growth and developmental processes as well as various biotic and abiotic stress responses in plants (Bari and Jones, 2009). For example, ABA is known to participate in various abiotic stresses, such as drought, cold, and osmotic stress (Finkelstein et al., 2002), and SA, JA and ethylene, are known to play important roles in regulating defense responses to pathogen infection (Glazebrook, 2005). GA signaling components also play key roles in plant disease resistance and susceptibility (Bari and Jones, 2009). We observed that the MdbZIP genes showed a range of response patterns when exposed to various hormone treatments, indicating that they may be involved in stress signaling. As mentioned in the results section, a similar conclusion was made for drought and salt stress, further suggesting that group A bZIP members participate in stress signaling, which is consistent with previous reports in *A. thaliana* (Jakoby et al., 2002). Gao et al. (2014) also suggested that most grape group A bZIP genes are induced by osmotic stress.

In conclusion, we performed the first genome-wide analysis of the apple bZIP TF family and conducted a detailed investigation of their structure and organization. We also characterized the expression of some group A genes following various hormonal and abiotic stresses. These results may prove useful in developing strategies for the future improvement of stress tolerance in apple.

## Author contributions

XW, JZ, and HG designed the study. JZ performed the experiments. JZ and RG and CG performed data analysis. XW and HG provided guidance on the whole study. JZ, HH, and XW Wrote and revised the manuscript. All authors approved the final manuscript.

### Conflict of interest statement

The authors declare that the research was conducted in the absence of any commercial or financial relationships that could be construed as a potential conflict of interest.
