# Supplementary material for: Evolutionary and Expression Analyses of the Apple Basic Leucine Zipper Transcription Factor Family
Source: Front Plant Sci. 2016 Mar 30;7:376. doi: 10.3389/fpls.2016.00376 (PMC4811886; doi:10.3389/fpls.2016.00376)
Supplement: Supplementary file 1 [file Table1.DOC]

**Supplementary Table S1** The 112 MdbZIP transcription factors in apple

| **Group** | **Gene** | **Locus ID** | **Chr** | **Start** | **End** | **Stand** | **CDS**  **(bp)** | **Peptide**  **(aa)** |
| --- | --- | --- | --- | --- | --- | --- | --- | --- |
| A(16) | MdbZIP94 | MDP0000169473 | chr15 | 17,793,553 | 17,794,938 | ＋ | 861 | 286 |
|  | MdbZIP70 | MDP0000636541 | chr12 | 20,963,304 | 20,970,848 | ＋ | 1455 | 484 |
|  | MdbZIP47 | MDP0000250947 | chr8 | 22,548,807 | 22,554,714 | － | 1563 | 528 |
|  | MdbZIP65 | MDP0000177486 | chr12 | 1,946,497 | 1,948,984 | － | 1394 | 470 |
|  | MdbZIP66 | MDP0000144105 | chr12 | 1,963,619 | 1,966,105 | ＋ | 1410 | 470 |
|  | MdbZIP23 | MDP0000231542 | chr4 | 8,917,835 | 8,924,220 | ＋ | 2982 | 1017 |
|  | MdbZIP57 | MDP0000740787 | chr10 | 29,978,607 | 29,983,162 | － | 1544 | 517 |
|  | MdbZIP5 | MDP0000273211 | chr2 | 30,554,835 | 30,560,990 | ＋ | 1522 | 535 |
|  | MdbZIP34 | MDP0000215106 | chr7 | 4,115,354 | 4,119,277 | － | 1206 | 408 |
|  | MdbZIP46 | MDP0000701734 | chr8 | 13,223,842 | 13,234,772 | － | 1857 | 643 |
|  | MdbZIP26 | MDP0000248567 | chr5 | 27,744,402 | 27,746,779 | － | 1295 | 431 |
|  | MdbZIP54 | MDP0000208334 | chr10 | 2,137,715 | 2,141,599 | － | 921 | 314 |
|  | MdbZIP98 | MDP0000129112 | chr15 | 40,776,203 | 40,779,181 | － | 1466 | 489 |
|  | MdbZIP48 | MDP0000706379 | chr8 | 27,911,843 | 27,914,522 | － | 870 | 299 |
|  | MdbZIP100 | MDP0000198495 | chr15 | 44,193,653 | 44,196,263 | － | 1147 | 391 |
|  | MdbZIP91 | MDP0000296303 | chr15 | 2,474,633 | 2,485,694 | ＋ | 2436 | 835 |
| B(5) | MdbZIP11 | MDP0000270677 | chr2 | 35,004,195 | 35,008,282 | － | 2262 | 755 |
|  | MdbZIP12 | MDP0000180785 | chr2 | 35,007,856 | 35,011,943 | ＋ | 2261 | 755 |
|  | MdbZIP29 | MDP0000299504 | chr7 | 1,372,519 | 1,376,449 | － | 2185 | 741 |
|  | MdbZIP31 | MDP0000378041 | chr7 | 1,391,809 | 1,395,238 | － | 246 | 100 |
|  | MdbZIP30 | MDP0000138811 | chr7 | 1,379,763 | 1,383,691 | ＋ | 2185 | 741 |
| C(6) | MdbZIP81 | MDP0000431572 | chr13 | 31,289,060 | 31,292,057 | ＋ | 1024 | 346 |
|  | MdbZIP82 | MDP0000441891 | chr13 | 31,289,069 | 31,292,136 | ＋ | 1018 | 346 |
|  | MdbZIP92 | MDP0000680042 | chr15 | 4,062,791 | 4,065,343 | ＋ | 965 | 327 |
|  | MdbZIP75 | MDP0000270365 | chr12 | 30,191,422 | 30,194,800 | － | 1283 | 437 |
|  | MdbZIP24 | MDP0000275309 | chr5 | 14,238,089 | 14,245,492 | － | 2331 | 799 |
|  | MdbZIP56 | MDP0000176747 | chr10 | 19,745,441 | 19,751,773 | － | 2,607 | 869 |
| D(16) | MdbZIP37 | MDP0000274723 | chr7 | 17,224,462 | 17,227,383 | － | 1239 | 416 |
|  | MdbZIP96 | MDP0000121258 | chr15 | 30,826,162 | 30,830,577 | － | 1551 | 524 |
|  | MdbZIP97 | MDP0000277999 | chr15 | 36,408,113 | 36,420,573 | ＋ | 2007 | 719 |
|  | MdbZIP85 | MDP0000320322 | chr14 | 7,564,687 | 7,571,368 | ＋ | 1607 | 542 |
|  | MdbZIP87 | MDP0000307943 | chr14 | 24,064,421 | 24,072,646 | － | 1410 | 494 |
|  | MdbZIP6 | MDP0000300532 | chr2 | 32,162,650 | 32,171,692 | － | 749 | 286 |
|  | MdbZIP76 | MDP0000145555 | chr13 | 2,703,862 | 2,707,043 | ＋ | 1,376 | 466 |
|  | MdbZIP101 | MDP0000250967 | chr16 | 1,376,596 | 1,386,527 | ＋ | 2980 | 1018 |
|  | MdbZIP77 | MDP0000301884 | chr13 | 2,710,089 | 2,713,271 | － | 1378 | 466 |
|  | MdbZIP33 | MDP0000320524 | chr7 | 3,433,485 | 3,446,567 | ＋ | 1572 | 526 |
|  | MdbZIP67 | MDP0000262210 | chr12 | 11,304,636 | 11,311,688 | ＋ | 1599 | 534 |
|  | MdbZIP108 | MDP0000536881 | chrun | 15,746,255 | 15,752,194 | ＋ | 1347 | 450 |
|  | MdbZIP18 | MDP0000488746 | chr3 | 5,626,620 | 5,631,464 | ＋ | 945 | 340 |
|  | MdbZIP17 | MDP0000301399 | chr3 | 5,626,357 | 5,631,201 | － | 963 | 340 |
|  | MdbZIP16 | MDP0000222114 | chr3 | 5,620,437 | 5,623,701 | ＋ | 821 | 280 |
|  | MdbZIP7 | MDP0000174930 | chr2 | 32,167,138 | 32,171,496 | ＋ | 849 | 306 |
| E(7) | MdbZIP51 | MDP0000178326 | chr9 | 32,583,190 | 32,585,878 | ＋ | 977 | 328 |
|  | MdbZIP52 | MDP0000306302 | chr9 | 32,586,980 | 32,588,028 | － | 816 | 272 |
|  | MdbZIP105 | MDP0000555457 | chr17 | 19,165,120 | 19,167,848 | － | 958 | 327 |
|  | MdbZIP111 | MDP0000123107 | chrun | 97,931,795 | 97,932,619 | ＋ | 690 | 229 |
|  | MdbZIP62 | MDP0000133698 | chr11 | 29,559,299 | 29,562,177 | ＋ | 1063 | 356 |
|  | MdbZIP19 | MDP0000134936 | chr3 | 27,871,861 | 27,873,934 | － | 1057 | 353 |
|  | MdbZIP112 | MDP0000267964 | chrun | 117,559,360 | 117,560,488 | － | 978 | 325 |
| F(6) | MdbZIP43 | MDP0000319187 | chr8 | 8,925,905 | 8,927,336 | － | 1076 | 361 |
|  | MdbZIP55 | MDP0000159670 | chr10 | 7,691,211 | 7,692,609 | － | 832 | 280 |
|  | MdbZIP32 | MDP0000772633 | chr7 | 1,913,688 | 1,914,425 | － | 731 | 245 |
|  | MdbZIP8 | MDP0000898701 | chr2 | 34,429,393 | 34,430,130 | － | 733 | 245 |
|  | MdbZIP9 | MDP0000893802 | chr2 | 34,458,341 | 34,459,078 | ＋ | 733 | 245 |
|  | MdbZIP10 | MDP0000190186 | chr2 | 34,462,771 | 34,463,508 | ＋ | 733 | 245 |
| G(8) | MdbZIP103 | MDP0000286846 | chr16 | 17,102,379 | 17,109,216 | ＋ | 1344 | 448 |
|  | MdbZIP27 | MDP0000185553 | chr6 | 3,270,543 | 3,277,761 | － | 1972 | 661 |
|  | MdbZIP89 | MDP0000231274 | chr14 | 28,607,551 | 28,610,272 | ＋ | 1054 | 351 |
|  | MdbZIP4 | MDP0000251332 | chr2 | 13,279,945 | 13,285,109 | ＋ | 1122 | 387 |
|  | MdbZIP109 | MDP0000545420 | chrun | 73,542,720 | 73,544,664 | ＋ | 830 | 278 |
|  | MdbZIP36 | MDP0000493795 | chr7 | 15,982,839 | 15,984,782 | ＋ | 827 | 278 |
|  | MdbZIP35 | MDP0000138052 | chr7 | 15,981,497 | 15,984,608 | ＋ | 1296 | 443 |
|  | MdbZIP1 | MDP0000197219 | chr1 | 10,520,899 | 10,523,983 | ＋ | 1357 | 457 |
| H(5) | MdbZIP68 | MDP0000586302 | chr12 | 17,051,287 | 17,053,624 | － | 477 | 164 |
|  | MdbZIP69 | MDP0000264514 | chr12 | 17,054,996 | 17,059,849 | ＋ | 669 | 233 |
|  | MdbZIP22 | MDP0000834642 | chr4 | 6,813,691 | 6,815,518 | － | 642 | 213 |
|  | MdbZIP79 | MDP0000279891 | chr13 | 10,470,937 | 10,474,463 | ＋ | 1073 | 366 |
|  | MdbZIP80 | MDP0000219041 | chr13 | 10,499,732 | 10,501,144 | － | 723 | 242 |
| I(18) | MdbZIP13 | MDP0000293847 | chr3 | 4,682,921 | 4,686,053 | ＋ | 1344 | 454 |
|  | MdbZIP14 | MDP0000120158 | chr3 | 4,782,005 | 4,784,925 | － | 1398 | 472 |
|  | MdbZIP15 | MDP0000295681 | chr3 | 4,782,814 | 4,784,704 | ＋ | 960 | 320 |
|  | MdbZIP59 | MDP0000297791 | chr11 | 4,673,815 | 4,676,378 | － | 1339 | 448 |
|  | MdbZIP60 | MDP0000305387 | chr11 | 4,683,517 | 4,686,081 | ＋ | 1335 | 448 |
|  | MdbZIP21 | MDP0000247372 | chr4 | 3,234,880 | 3,237,744 | ＋ | 1265 | 421 |
|  | MdbZIP110 | MDP0000129203 | chrun | 79,664,124 | 79,666,821 | ＋ | 1077 | 361 |
|  | MdbZIP74 | MDP0000210251 | chr12 | 26,145,161 | 26,147,824 | ＋ | 1057 | 361 |
|  | MdbZIP93 | MDP0000435971 | chr15 | 11,167,017 | 11,170,907 | － | 1304 | 449 |
|  | MdbZIP58 | MDP0000234166 | chr11 | 1,063,026 | 1,065,436 | ＋ | 1038 | 345 |
|  | MdbZIP106 | MDP0000120802 | chrun | 7,122,677 | 7,124,802 | ＋ | 1318 | 439 |
|  | MdbZIP71 | MDP0000280559 | chr12 | 21,390,957 | 21,393,913 | － | 1741 | 580 |
|  | MdbZIP72 | MDP0000479652 | chr12 | 21,395,646 | 21,398,279 | － | 1584 | 527 |
|  | MdbZIP38 | MDP0000282828 | chr8 | 1,209,801 | 1,216,496 | － | 3459 | 1166 |
|  | MdbZIP39 | MDP0000300820 | chr8 | 1,214,016 | 1,216,474 | － | 1519 | 1519 |
|  | MdbZIP99 | MDP0000602946 | chr15 | 41,411,070 | 41,414,903 | － | 1258 | 421 |
|  | MdbZIP53 | MDP0000141948 | chr10 | 1,902,910 | 1,910,401 | － | 2695 | 917 |
|  | MdbZIP25 | MDP0000236212 | chr5 | 18,793,406 | 18,793,794 | ＋ | 309 | 103 |
| J(2) | MdbZIP83 | MDP0000121603 | chr14 | 1,336,067 | 1,339,625 | － | 1527 | 514 |
|  | MdbZIP84 | MDP0000147745 | chr14 | 1,336,237 | 1,339,584 | － | 1755 | 588 |
| S(21) | MdbZIP42 | MDP0000891108 | chr8 | 6,132,321 | 6,132,899 | ＋ | 578 | 192 |
|  | MdbZIP102 | MDP0000183562 | chr16 | 1,860,154 | 1,861,757 | － | 1076 | 360 |
|  | MdbZIP78 | MDP0000891899 | chr13 | 3,386,464 | 3,387,078 | － | 614 | 204 |
|  | MdbZIP50 | MDP0000407755 | chr9 | 4,760,296 | 4,760,904 | ＋ | 608 | 202 |
|  | MdbZIP104 | MDP0000863909 | chr17 | 5,338,915 | 5,339,523 | ＋ | 605 | 202 |
|  | MdbZIP28 | MDP0000917315 | chr6 | 22,353,119 | 22,353,583 | ＋ | 465 | 154 |
|  | MdbZIP88 | MDP0000905135 | chr14 | 26,993,073 | 26,993,594 | ＋ | 521 | 173 |
|  | MdbZIP2 | MDP0000249561 | chr2 | 12,516,047 | 12,516,520 | － | 474 | 157 |
|  | MdbZIP3 | MDP0000265875 | chr2 | 12,713,645 | 12,714,118 | － | 473 | 157 |
|  | MdbZIP41 | MDP0000205823 | chr8 | 2,303,356 | 2,303,835 | ＋ | 480 | 159 |
|  | MdbZIP61 | MDP0000239026 | chr11 | 24,590,229 | 24,590,711 | － | 483 | 160 |
|  | MdbZIP40 | MDP0000521934 | chr8 | 1,641,214 | 1,641,684 | ＋ | 463 | 156 |
|  | MdbZIP73 | MDP0000448715 | chr12 | 21,746,621 | 21,747,094 | ＋ | 474 | 157 |
|  | MdbZIP20 | MDP0000437680 | chr3 | 33,166,561 | 33,166,989 | － | 428 | 142 |
|  | MdbZIP63 | MDP0000234798 | chr11 | 34,951,292 | 34,951,717 | ＋ | 426 | 141 |
|  | MdbZIP64 | MDP0000949327 | chr11 | 34,954,027 | 34,954,452 | － | 426 | 141 |
|  | MdbZIP44 | MDP0000140166 | chr8 | 11,589,213 | 11,589,767 | ＋ | 554 | 184 |
|  | MdbZIP45 | MDP0000200822 | chr8 | 11,591,288 | 11,591,746 | － | 459 | 152 |
|  | MdbZIP90 | MDP0000190277 | chr15 | 1,339,936 | 1,340,394 | － | 459 | 152 |
|  | MdbZIP49 | MDP0000738631 | chr8 | 28,097,624 | 28,098,310 | ＋ | 684 | 228 |
|  | MdbZIP95 | MDP0000261154 | chr15 | 20,032,093 | 20,041,062 | － | 2540 | 873 |
| Un(2) | MdbZIP107 | MDP0000386314 | chrun | 9,106,458 | 9,114,241 | － | 1302 | 433 |
|  | MdbZIP86 | MDP0000239688 | chr14 | 14,476,695 | 14,489,099 | ＋ | 2564 | 854 |
