# Supplementary material for: Evolutionary and Expression Analyses of the Apple Basic Leucine Zipper Transcription Factor Family
Source: Front Plant Sci. 2016 Mar 30;7:376. doi: 10.3389/fpls.2016.00376 (PMC4811886; doi:10.3389/fpls.2016.00376)
Supplement: Supplementary file 3 [file Table3.DOC]

**Supplemental Table S3: Additional conserved motifs identified from 112 MdbZIP proteins**

| **Motif No.** | **Length (aa)** | **E-value** | **Multi-level Consensus Sequences** | **No. of MdbZIP proteins in which motif is present** | **MdbZIP proteins in which motif is present** |
| --- | --- | --- | --- | --- | --- |
| 1 | 29 | 8.9e-1788 | [EP][KR][RK]Q[KR]R[MIL][LI][SA]NR[EQ]SA[RA][RK]SR[EL]RK[QK]A[YH][VI]xELE | 112 | all MdbZIPs |
| 2 | 143 | 1.4e-859 | GA[LT]TF[DEN][MLV]EY[AG][RH]WLEE[HQ][NHQ]R[QL]I[CHN][ED][LM]R[SA]A[VL]NSH[AM][GS]DNELRILVD[NS][VG][ML][AT]HYDE[IL]FR[LM]K[GS][TI]AAK[AT]DVF  [HY][IM][LV]SGMWKT[PS]AERCFMW[IL]GGFR[SP]SELLK[IL]L[GLV][NP][QH]LEPLT  [DE]QQL[LMV][GD]ICNL[QR]QSSQQAEDALSQGM[ED][AK]LQQSL[ASV]ET[LI][AS][SA][GD][SQ][LH][CGS] | 11 | MdbZIP 33,37,67,76,77,85  87,96,97,101,108 |
| 3 | 63 | 1.5e-484 | RKVQTLQ[TS]E[AI]TTLS[AP]Q[LVF]T[LF][FL]Q[RH][DQ]TTGL[ST][ST]EN[TK]ELKLR[LI][QA][AS][ML]EQ[QD][AK]QL[RK]DALN[ED]ALKKEV[EQ]RL[KR] | 18 | MdbZIP13,14,16,17,18,19,21,38,53,59,60,62,71,72,74,93,99,110 |
| 4 | 200 | 4.4e-367 | IPKPGGNRGIHA[PS]LYR[NS]SAGPHKALT[AS]GSTDVPKD[HQ]MKST[ST]ADGKLQQWFREGLAGPMLSSGMCTEVFQFDVSAATSSGGIIPASSVSN[AV]SEHRQNTTELNRGRNRRIL[HR]GLPIPL[AP]GSGHNVTEENV[KT]GNQQNNTLP[DG][NS]RSVSPMVVSVLVDPREAGDIDVDGMIKPKSLSRIFVVVLLDSVKYVTYSCVLPRSGPHLV | 4 | MdbZIP 11,12,29,30 |
| 5 | 200 | 7.2e-354 | HPPMPPM[AP]YPWMPYSPYV[TV][KN]PQGSQSLL[LV]PIPRLKSQQPAAAPKS[KR]KS[DE]SKKSEGRTKKVAS[IV]SFLGLLFFILLFGGLVPMVNVNFS[DG]VMDRGFGGSSY[AV][HS]DRFNGQ[NY]RGRLLNVHGHLN[AG]SEEII[GS]SGISGGKFDVSNK[IV][LR]HE[KR]GHHTRN[GM]PKEQGSR[AS][TV]PGSDEFARLHN[IT]SSEPLVASLYVPRN[DE]KLVKIDGNLIIH | 4 | MdbZIP 11,12,29,30 |
| 6 | 159 | 1.2e-354 | [FA][NT][SF][NF][PS][NM][PQ][NL]P[NM]P[KP][SP]N[PG]I[PY][PA][LW][PG][RG]SVGI[DS]GNANASGLAAAATSSSSTS[FS]TTSTSLSLATMLGNSASPFIREDADGSH[HL][DH]H[ND][HY][DG][VG]SHRRA[NH]SEVSFRL[PS][KE]DM[MV]D[LM][PS]AAA[GA]DPFNGGSST[PA]SLEEMGSEEDLFSTYIDLDKLGGSNNSSDQN[SG]GGNDGP[DN]GGPN | 5 | MdbZIP 13,14,15,59,60 |
| 7 | 57 | 9.7e-334 | M[GE]KL[AEG][TA]LE[GSN]F[LV][HR]QAD[NHL]LRQQTLQQ[ML][HS]RILTTRQ[AS]AR[AG]LL[AV][IL][GS][DE]YF[SH]RLRALSSLW[LQ]A | 11 | MdbZIP 33,37,67,76,77,85,87,96  97,101,108 |
| 8 | 21 | 1.6e-222 | [EQ]VxQLRxENSQLKx[RQ]LxxLxQ | 77 | all except MdbZIP 6，7，13，14，16，17，18，19，21，25，31，33，37，53，59，60，62，67，71，72，74，76，77，85，87，93，96，97，98，99，101，108，110，111，112 |
| 9 | 48 | 1.7e-167 | L[AG]RQ[SN]SIYSLT[LF][DE]E[FLV]Q[NH][TQ][LI][GC][EGD][SL]GK[DNP][FL][GS]SMN[ML]DE[LF]L[KT][SN][IV]W[TS]AE[EA][NT]Q[AI][IM][NAG] | 8 | MdbZIP 5，23，26，34，46，65，66，91 |
| 10 | 141 | 7.5e-137 | VHMPLA[DE]ASA[DE]K[HS]IATQPSLPRQGSLTLP[AE]PLCRKTVDEVWS[VE]I[HN]KGQQAKQQNNHN[GS]SIDGGVQSSEFAPRQPTFGEMTLEDFLVKAGVVREQDSMAATVVPPQP[QH]QQQ[QR]YGMYQNGN[QH]AV[VG]PSFVNRPVMGMGAA[GA]AAG | 3 | MdbZIP 23，65，66 |
| 11 | 41 | 4.3e-147 | [STD][APT][GRH][TVA][CT]T[HV][TL][HE][TL][CN]NPP[GI][PL][DN][ASY][AKS][HT]T[HP]TC[YVF][HP][TPV][HS][TC][QAK][IV]L[SP][SP][EA][DTS][DWE][DI][DGK][TAN][DK] | 9 | MdbZIP 8，9，10，32，35，36，43，55，109 |
| 12 | 29 | 6.2e-137 | [ST]SRLKL[TV]QLEQELQRARQQG[IV][FY][IL][SG]G[GS][LG][DG][GQ] | 16 | MdbZIP 6，7，16，17，18，33，37，67，76，77，85，87，96，97，101，108 |
| 13 | 23 | 2.0e-133 | [FYH]DN[VAI]E[ST][ED]N[ARD][VR]L[KR][ASE][DEQ][AV][ES][EA]LR[AQ][RKM][LV][KQS][DS][LA][ENQ]EI[LVS] | 25 | MdbZIP20,24,28,40,41,42,44,45,49,50,56,61,63,64,73,75,78,81,82,88,90,92,95,102,104 |
| 14 | 99 | 1.9e-129 | Q[LT][IS][PL][ADV][GR][RS][RA][TS][SG][LFR]D[EGN]S[AG][SI][GA]KP[VIN]QLM[KR]QGSNGNAEG[MV]D[ED]RKSGGE[VI][VA]DDLFNAYMNLENID[QK]MN[LS]SGNEDKD[MIL]DSRAS[GA]SKTNG[GC]ESSDNEVESGL[NK]GN[RG]NG | 4 | MdbZIP 38,39,71,72 |
| 15 | 21 | 7.9e-117 | [NE][AV][IM][ED][AL]KK[AI]M[APE][PN][DE]KLAE[LI][WA][LT][LSV]D | 17 | MdbZIP13,14,15,21,38,39,53,58,59,60,71,72,74,93,99,106,110 |
| 16 | 58 | 4.1e-115 | [QT]K[MT]PPS[MA]GGCFAPNGNAI[AQ][AQ][TQ][TQ]SQ[NS]P[SN]WVDEFLDFSSARRG[SA]HRRS[IA]SDSI[TA]FLE[AS]P[ML] | 6 | MdbZIP 19,51,52,62,105,111 |
| 17 | 112 | 5.9e-170 | D[EP]FMSDLGFGFG[DP]D[DG]NC[DE]FELTFDDLD[DK]LYLPS[EV][AT]EDFL[LV]PDGLDP[GS]AA[AE][LS]NSGSPESGSSAI[MS]VSGDDKG[AG]LD[IV]SRFLNCPASSNECSENS[DG]GPASSQGSGISEAVDS[HN]S[GH] | 4 | MdbZIP 11,12,29,30 |
| 18 | 29 | 1.1e-103 | [QS][RA][QT][LQ][TK]L[EG][EN][MT]TL[EL]D[FK]L[VK][RKN]A[GR][AV][VG]R[EM][ED]D[IQ][IKS][MS]N | 14 | MdbZIP1,5,26,34,35,36,46,48,54,57,91,98,100,109 |
| 19 | 37 | 7.5e-094 | S[AP][YF]FP[LS]Q[QS]Q[PT]G[PS][AR][SD][HS]Q[TN][MI][HQ][LM][PR]QF[NH][HQ][SF]Q[SP][NS]M[PS][TA][QP]H[LQ][HP][QM] | 7 | MdbZIP 13,14,21,59,60,74,110 |
| 20 | 80 | 1.4e-141 | ID[TN]ELG[VA]FPF[GQ][NK]Q[CL]NSNTYF[EK][DE]SQCNLQSSVGAMGL[RH]CQTDLPC[FS]CPPVGSSVQA[SG]IGAR[GQ][EK]TMV[PS]SG[GR]NCQPAVIDCRA | 4 | MdbZIP 8,9,10,32 |
| 21 | 21 | 4.1e-087 | [GR][GT]A[GS]G[DG][EV][KV][PR]RSRH[RC][HRY]S[SI]S[MV]D[GS] | 17 | MdbZIP13,14,15,21,38,39,53,58,59,60,71,72,74,93,99,106,110 |
| 22 | 65 | 9.3e-078 | R[PS]QS[GR]SWQ[LM]G[QT]QN[LF]S[SP]GSSH[TA]RS[ML]SQPP[FV]FSLDSLPPLSPLTYR[ED]PS[PAG]PS[LP]SD[PQ]NSV[DE]VSM[GE]E[ST]V | 4 | MdbZIP 38,39,71,72 |
| 23 | 200 | 3.6e-072 | KVLDSCGSDIIELGVPYSDPLADGPVIQAAATRSLARGTNLNAIISMLKEVIPQLSCPIALF[AT]YYNPILKRGIPEFMSTIKDVGVHGLVVPDVPLEETEILRKEA[AV]KNNIELVLLTTPTTPIDRMKAIAEASEGFLYLVSSIGVTGARASVNERVPALLKEIKETTK[KR]PVAVGFGISKPEHAKQVAGWGADGVIVGSAMV | 2 | MdbZIP24,56 |
| 24 | 200 | 3.6e-072 | FCLFNRPPITPLFWPxIIQSSNSIQVQHVLQNPIAVLSNISLPANGAAxSSLDQETPPNINGARTPLCVFPCPWFIPHFDNGxGLQPQSSLCLNNKQEExSFNNQYSSSSSSRAxTQLDNHQSSLPVRLKTQ  DSASIEGRPSHDLNETPAQFPLDEGDQHTGPHPKENGCKEIFLSPSSLNHAGVASCIKHENGF  EPDHT | 2 | MdbZIP 83,84 |
| 25 | 159 | 7.1e-063 | VHSQTASSPFELKREFSLGEALEYLMDKSMIGQEGADTQGHQxEKMDEYWAKYRNFASADVFxGDVCAGDLSFAFKNEDIMDGFSSFGELTETLLCTQNLTPKNSCISATMDSQSSICVGCPTSAAKPIGRDNQARGAYSGSSGEQSDEDDFEMEFGPC | 2 | MdbZIP 81,82 |
